# Gene fusions and oncogenic mutations in MLH1 deficient and *BRAF*V600E wild-type colorectal cancers

**DOI:** 10.1007/s00428-022-03302-x

**Published:** 2022-03-03

**Authors:** Iiris Ukkola, Pirjo Nummela, Mia Kero, Hanna Tammio, Jenni Tuominen, Veli Kairisto, Markku Kallajoki, Caj Haglund, Päivi Peltomäki, Soili Kytölä, Ari Ristimäki

**Affiliations:** 1 Department of Pathology, HUSLAB, HUS Diagnostic Center, Helsinki University Hospital and University of Helsinki, P.O. Box 400, 00029 HUS, Helsinki, Finland; 2 Applied Tumor Genomics Research Program, Research Programs Unit, Faculty of Medicine, University of Helsinki, Helsinki, Finland; 3 Department of Genetics, HUSLAB, HUS Diagnostic Center, Helsinki University Hospital and University of Helsinki, Helsinki, Finland; 4 Department of Genomics, Laboratory of Molecular Haematology and Pathology, Turku University Central Hospital, Turku, Finland; 5 Department of Pathology, University of Turku and Turku University Hospital, Turku, Finland; 6 Department of Surgery, University of Helsinki and Helsinki University Hospital, Helsinki, Finland; 7 Translational Cancer Medicine Research Program, Research Programs Unit, Faculty of Medicine, University of Helsinki, Helsinki, Finland; 8 Department of Medical and Clinical Genetics, University of Helsinki, Helsinki, Finland

**Keywords:** *ALK*, *BRAF*, Colorectal cancer, Gene fusion, Mismatch repair, *NTRK*, *RET*

## Abstract

Gene fusions can act as oncogenic drivers and offer targets for cancer therapy. Since fusions are rare in colorectal cancer (CRC), their universal screening seems impractical. Our aim was to investigate gene fusions in 62 CRC cases with deficient MLH1 (dMLH1) and *BRAF*V600E wild-type (wt) status from a consecutive real-life series of 2079 CRCs. First, gene fusions were analysed using a novel FusionPlex Lung v2 RNA–based next-generation sequencing (NGS) panel, and these results were compared to a novel Idylla GeneFusion assay and pan-TRK immunohistochemistry (IHC). NGS detected seven (7/62, 11%) *NTRK1* fusions (*TPM3::NTRK1*, *PLEKHA6::NTRK1* and *LMNA::NTRK1*, each in two cases, and *IRF2BP2::NTRK1* in one case). In addition, two *ALK*, four *RET* and seven *BRAF* fusions were identified. Idylla detected seven *NTRK1* expression imbalances, in line with the NGS results (overall agreement 100%). Furthermore, Idylla detected the two NGS–identified *ALK* rearrangements as one specific *ALK* fusion and one *ALK* expression imbalance, whilst only two of the four *RET* fusions were discovered. However, Idylla detected several expression imbalances of *ALK* (*n* = 7) and *RET* (*n* = 1) that were found to be fusion negative with the NGS. Pan-TRK IHC showed clearly detectable, fusion partner-dependent staining patterns in the seven *NTRK1* fusion cases. Overall agreement for pan-TRK antibody clone EPR17341 was 98% and for A7H6R 100% when compared to the NGS. Of the 62 CRCs, 43 were *MLH1* promoter hypermethylated (*MLH1*ph) and 39 were *RAS*wt. All fusion cases were both *MLH1*ph and *RAS*wt. Our results show that kinase fusions (20/30, 67%) and most importantly targetable *NTRK1* fusions (7/30, 23%) are frequent in CRCs with dMLH1/*BRAF*V600Ewt/*MLH1*ph/*RAS*wt. NGS was the most comprehensive method in finding the fusions, of which a subset can be screened by Idylla or IHC, provided that the result is confirmed by NGS.

**Supplementary Information:**

The online version contains supplementary material available at 10.1007/s00428-022-03302-x.

## Introduction

Universal screening for mismatch repair deficiency (dMMR) amongst colorectal cancer (CRC) patients has been recommended to facilitate identification of Lynch syndrome (LS) and to direct optimal oncological treatment of those cases presenting a sporadic microsatellite unstable tumour [1]. For the treatment of adult patients with unresectable or metastatic CRC with dMMR or microsatellite instability (MSI), European Medicines Agency (EMA) has approved first-line monotherapy treatment of immuno-oncological drug pembrolizumab in 2020. Besides DNA-repair deficiency phenotype in CRC, gene fusions that act as oncogenic drivers offer targets for cancer therapy. To this end, larotrectinib became the first and entrectinib the second tumour agnostic, i.e. ‘histology-independent’, cancer treatment approved by EMA (2019 and 2020, respectively) in patients whose solid tumours display a *neurotrophic tyrosine receptor kinase* (*NTRK*) gene fusion and are advanced, have spread to other parts of the body or are not amenable to surgery, and who have no satisfactory alternative treatments [2]. The family of *NTRK* genes consists of *NTRK1-3* encoding TRKA, TRKB and TRKC proteins that play a role in development and functioning of the nervous system, and act as drivers of oncogenesis in various cancers [3]. Only 0.2–0.3% of CRCs harbour *NTRK* gene fusions, which makes universal screening of CRC patients for this gene rearrangement impractical. However, recent studies have recognised an enrichment of *NTRK* fusions in a subset of CRCs presenting dMMR due to loss of *MLH1* gene expression, *BRAF*V600E wild-type (wt), *MLH1* promoter hypermethylation (*MLH1*ph) and *RAS*wt [4–7].

Gene fusions can be studied using DNA–, RNA– or combined DNA/RNA–based next-generation sequencing (NGS), as well as with fluorescence in situ hybridization (FISH), reverse transcription-polymerase chain reaction (RT-PCR) and immunohistochemistry (IHC). ESMO recommendations propose that IHC can be used as a screening method (if no smooth muscle or neuronal differentiation is present) to enrich patients with *NTRK* fusions in an unselected population [2, 8]. FISH and RT-PCR are recommended to be used in tumour types that harbour high frequency of a specific *NTRK* fusion, such as *ETV6::NTRK3*, which is relatively infrequently found in CRC. DNA– and/or RNA–based NGS panels can be used either upfront or to confirm the presence of *NTRK* fusion in TRK immunopositive tumours or those devoid of other driver mutations, such as those in *BRAF* and *RAS* genes. Of the NGS platforms, RNA–based panels are favoured due to their ability to detect both known and novel fusions and higher sensitivity when compared to the DNA–based ones. Recently, a novel fully automated quantitative RT-PCR option has been introduced, the research use only (RUO) Idylla gene fusion assay, which can detect several oncogenic gene fusions.

The aim of this study was to investigate enrichment of *NTRK* and other oncogenic gene fusions in a cohort of dMLH1 and *BRAF*V600Ewt CRC cases (*n* = 62), which originated from universal dMMR screen of over two thousand consecutive CRC patients in a real-life diagnostic setting. First, gene fusions were analysed using a novel RNA–based FusionPlex Lung v2 NGS panel, and these results were then compared to a novel RNA–based Idylla GeneFusion assay and pan-TRK immunohistochemistry (IHC). In addition to MMR and *BRAF* mutation status, all 62 cases were analysed for MSI, *MLH1*ph and *RAS* mutation status.

## Materials and methods

### Sample selection

Universal dMMR screening for all newly diagnosed CRC cases has been routine in Helsinki University Hospital (HUH) since January 2018, using IHC for MMR proteins MLH1, MSH2, MSH6 and PMS2. In case of deficient MLH1 IHC, the dMMR screening algorithm leads to BRAFV600E mutation–targeted IHC testing to identify potential LS patients, i.e. immunonegative and thus BRAFV600Ewt, to be further tested for *MLH1*ph (methylation-positive cases interpreted as sporadic dMLH1). Our patient material consisted of consecutive CRC patients undergoing primary surgery at HUH between January 2018 and April 2020 (*n* = 2079), out of which 66 showed immunohistochemically confirmed loss of expression of MLH1 (and concomitant loss of PMS2) and BRAFV600Ewt status. Two of the 66 samples were not representative for our study and two were not available leading to a study cohort of 62 samples. The study was approved by the Ethics Committee of the HUH.

### MLH1 promoter hypermethylation analysis

*MLH1* methylation status of the 62 CRC cases was determined with methylation-specific multiplex ligation-dependent probe amplification (MS-MLPA). Here, SALSA MS-MLPA Probemix ME011-D1 Mismatch Repair Genes kit (MRC Holland, Amsterdam, the Netherlands) was used to determine the methylation status of the promoter regions of MMR genes (*MLH1, MSH2, PMS2* and *MSH6*) using probes that contain a digestion site for the methylation-sensitive restriction endonuclease HhaI. At the same time, the *BRAF*V600E point mutation was detected with a probe specific for this mutation. The kit includes six probe pairs for *MLH1* (covering A to D sites in *MLH1* promoter and *MLH1* intron 1 93 nt after exon 1). All reactions and analysis of the results were accomplished according to the manufacturer’s instructions and as described in Gylling et al. [9]. DNA amount of 250 ng extracted from formalin-fixed paraffin-embedded (FFPE) tissue samples was used for each MLPA reaction. As a threshold, ratio of > 0.15 (corresponding to > 15% of methylated DNA) was used to indicate promoter methylation. In addition, 45/62 CRC cases were analysed using bisulphite pyrosequencing of the *MLH1* promoter region to detect methylated cytosines in tumour DNA at positions c.-269, c.-262, c.-252 and c.-250 of the *MLH1* gene, showing 100% concordant results.

### Idylla KRAS and NRAS-BRAF mutation tests, and MSI test

*KRAS* and *NRAS* mutations were identified, and the *BRAF*V600wt status confirmed, from the 62 CRC FFPE samples using real-time PCR–based CE-IVD–validated Idylla KRAS and NRAS-BRAF mutation assays (Biocartis NV, Mechelen, Belgium). Furthermore, the MSI status of the dMMR samples was confirmed with Idylla MSI test that was performed as described previously [10]. The Idylla KRAS Mutation Test detects 21 *KRAS* mutations in exons 2, 3 and 4, whereas the Idylla NRAS-BRAF Mutation Test detects 18 *NRAS* mutations in exons 2, 3 and 4, and five *BRAF* mutations in codon 600. For the analyses, 10-µm thick tissue slices were cut from the CRC tissue blocks. The tissue sample handling and analysis were performed according to the manufacturer’s protocol and 20 to 80% of tumour cell content was used.

### Next generation sequencing (NGS) analysis

The cohort of 62 dMLH1/*BRAF*V600Ewt CRC FFPE samples underwent RNA–based targeted NGS analysis using novel RUO FusionPlex® Lung v2 (Invitae Corporation, San Francisco, CA) which identifies fusion transcripts of *ALK, BRAF, EGFR, ERBB2, FGFR1, FGFR2, FGFR3, KRAS, MET, NRG1, NTRK1, NTRK2, NTRK3, NUTM1, PIK3CA, RET* and *ROS1* genes. Total nucleic acid was extracted from 10-µm FFPE tissue sections using QIASymphony RNA Kit (QIAGEN, Valencia, CA), and the quality was assessed by Qubit RNA HS Assay Kit (Thermo Fisher Scientific, Waltham, MA). RNA was then reversely transcribed, and the quality was checked by ABI StepOne Plus (Applied Biosystems/Thermo Fisher Scientific). Libraries were quantified with KAPA Library Quantification Kit Illumina® Platforms (ABI StepOne Plus). Libraries were paired-end sequenced at 2 × 150 cycles on Illumina Novaseq 6000 instrument using SP flow cell with Novaseq Xp workflow (Individual Lane loading). Data were analysed using the Archer Analysis v6.2.7 software (Archer/Invitae) for the presence of gene fusion using GRCh37 as the reference genome (Supplementary Table S1).

*NTRK1* fusions were further validated with two independent RNA–based NGS platforms, FusionPlex Comprehensive Thyroid and Lung panel (CTL AK0070 v1.1, Invitae) and TruSight RNA Pan-Cancer panel (Illumina, San Diego, CA). FusionPlex CTL Kit detects fusion transcripts of *ALK, AXL, BRAF, CCND1, FGFR1, FGFR2, FGFR3, MET, NRG1, NTRK1, NTRK2, NTRK3, PPARG, RAF1, RET, ROS1* and *THADA* genes. In short, total nucleic acid was extracted from 10-µm FFPE tissue sections using QIASymphony RNA Kit (QIAGEN), and the quality was assessed by Qubit RNA HS Assay Kit (Thermo Fisher Scientific). RNA was then reversely transcribed, and the quality was checked by ABI StepOne Plus (Applied Biosystems/Thermo Fisher Scientific). Libraries were quantified with Ion Library TaqMan Quantitation Kit (ABI StepOne Plus) and sequenced on either Ion S5 or Ion Proton systems (IonTorrent/Thermo Fisher Scientific). Data were analysed using the Archer analysis software (Suite_Analysis_v6.0.4; Invitae) for the presence of gene fusion using GRCh37 as the reference genome (Supplementary Table S1).

For Illumina TruSight RNA Pan-Cancer panel, total RNA was extracted from 10-µm FFPE tissue sections using RNeasy FFPE kit (QIAGEN) according to the manufacturer’s protocol. The RNA quality (DV200) was assessed using the Agilent Bioanalyzer 2100 instrument (Agilent, Santa Clara, CA). Targeted RNA libraries were prepared according to the Illumina TruSight RNA Pan-Cancer panel reference guide (Illumina), and the amount of input RNA was evaluated based on DV200 value. The final libraries were paired-end sequenced at 2 × 75 cycles on Illumina MiniSeq instrument using its High Output kit. Illumina DRAGEN software (v3.8.4) was used for the fusion calling and GRCh38 was used as the reference genome (Supplementary Table S1).

NGS analysis of oncogenic mutations in a metastasized case (#52) with synchronous CRC was done using an in-house cancer panel containing seven target genes (*PIK3CA*, *EGFR*, *KIT*, *KRAS*, *MET*, *NRAS* and *PDGFRA*) and exons 11–15 of *BRAF* and was performed as previously described [11].

### Idylla gene fusion test

All 62 cases were further tested with the RUO real-time RT-PCR–based Idylla™ GeneFusion Assay (Biocartis NV) to detect *ALK*, *ROS1*, *RET* and *MET* exon 14 skipping and *NTRK*1/2/3 fusions in single cartridge and approximately in 3 h. For *NTRK1/2/3*, *ALK*, *ROS* and *RET* fusions, the detection was performed with expression imbalances indicating putative gene fusion irrespective of fusion partner based on the 3’ kinase overexpression caused by the partner gene. For *ALK, ROS* and *RET* fusions and *MET ex14* skipping, the detection was additionally performed by assessing specific gene fusion variations of the most common variants. For the analysis, 10-µm sections were cut from the FFPE CRC tissue blocks. The tissue sample handling and analysis were performed according to the manufacturer’s protocol and 20 to 80% of tumour cell content was used.

### Immunohistochemistry

For immunohistochemical analyses, 4-µm sections cut from the FFPE CRC tissue blocks (*n* = 62) were used. The MMR IHC was performed as described previously [10] and BRAFV600E mutation was detected by using the specific monoclonal ready-to-use antibody (clone VE1, 760–5095, Roche, Tucson, AZ), utilising detection with OptiView DAB kit (760–700, Roche) and additional Amplification Kit (760–099, Roche) [12]. Pan-TRK IHC was performed using ready-to-use monoclonal antibody (clone EPR17341, 790–7026, Roche) and detection by OptiView DAB kit (760–700, Roche). ALK IHC was performed using monoclonal antibody (clone 5A4, Novocastra™, Leica Biosystems, Newcastle Upon Tyne, UK) at dilution 1:50 and Roche’s OptiView DAB and Amplification kits. All above-described immunostainings were performed with the Ventana Benchmark ULTRA immunostainer (Roche). The other pan-TRK antibody used in this study (clone A7H6R, #92,991, Cell Signaling Technology Inc., Danvers, MA) was diluted 1:50 and stained with Autostainer (Agilent) using Envision Flex High pH detection kit (K8000, Agilent). Each slide had a non-neoplastic colorectal specimen as a positive external control (neural structures), and since we used (freshly cut) whole tissue sections of the tumour samples, most of them also contained positive internal control (neural structures in the muscularis propria). IHC stainings were analysed in a blinded manner by IU and AR. Positive pan-TRK immunoreactivity, indicating putative *NTRK* fusion, was determined when ≥ 1% of the cancer cells displayed cytoplasmic, membranous, nuclear and/or perinuclear immunopositivity.

### Statistical analysis

The RNA–based NGS panel (FusionPlex Lung v2) was considered as the gold standard test against which the overall agreement, sensitivity and specificity, and the positive and negative predictive values (PPV and NPV, respectively) were calculated. Fisher’s exact test (GraphPad QuickCalcs: https://www.graphpad.com/quickcalcs/contingency2/) was used for comparison of the agreement between NGS and the Idylla test, and between NGS and the IHC methods. Clinicopathological characteristics between fusion positive and negative cases were compared using Mann–Whitney *U*-test (numerical variables) or Fisher’s exact test (categorical variables). All statistical tests were two-tailed and numeric variables are reported by median and range. *P*-value less than 0.05 was considered as statistically significant.

## Results

### Clinicopathological characteristics and MLH1 promoter hypermethylation and RAS mutation status of the CRC cases

Our study consisted of 62 CRC samples with dMLH1 and BRAFV600Ewt status as detected by IHC. Of these patients, 59.7% were females and the tumours localised mainly to the right colon (85.5%), were low-grade (69.4%), pT3 (59.7%), pN0 (64.5%) and M0 (80.6%) (Table 1). Of the cases, 42/60 were *MLH1*ph as detected by MS-MLPA. One case was not available for the analysis. One case was not analysable despite three attempts, but was successfully analysed using bisulphite pyrosequencing and found to be *MLH1*ph. Overall, 43/61 (70.5%) of the CRC cases were *MLH1*ph (Supplementary Table S2). *KRAS* mutations were detected in 20 cases by Idylla KRAS mutation test, and three *NRAS* mutations were detected by Idylla NRAS-BRAF mutation test (Supplementary Table S2**)**. Overall, *RAS* mutations were found in 23 (37.1%) of the dMLH1/BRAFV600Ewt CRC cases. *KRAS* mutations were the most common in codon 12 (10 cases), followed by codon 61 (five), codon 146 (four) and codon 13 (one). *NRAS* mutations were found in codons 61 (two cases) and 12 (one case). MSI status was verified using Idylla MSI test, which resulted MSI in 62/62 of the cases (one sample, case 16, was initially MSS but after re-analysis using a more representative tissue block resulted MSI). Finally, the NRAS-BRAF test (*n* = 62) and the MS-MLPA (*n* = 60) analyses confirmed the *BRAF*V600Ewt status of all CRC cases.

**Table 1:** Characteristics of MLH1 deficient and *BRAF*V600E wild-type CRC cases (*n* = 62)

Age (years)	Median (range)	71 (36–91)
Sex	Female	37 (59.7%)
	Male	25 (40.3%)
Tumour site	Left colon	9 (14.5%)
	Right colon	53 (85.5%)
Grade	Low-grade	43 (69.4%)
	High-grade	19 (30.6%)
pT	T1	3 (4.8%)
	T2	10 (16.1%)
	T3	37 (59.7%)
	T4a	6 (9.7%)
	T4b	6 (9.7%)
pN	N0	40 (64.5%)
	N1a	5 (8.1%)
	N1b	4 (6.5%)
	N1c	2 (3.2%)
	N2a	6 (9.7%)
	N2b	5 (8.1%)
M	M0	50 (80.6%)
	M1a	5 (8.1%)
	M1b	3 (4.8%)
	M1c	4 (6.5%)

*CRC* colorectal cancer, *T* tumour, *N* nodes, *M* metastases

### RNA–based NGS fusion panel analysis

**Fig. 1:**
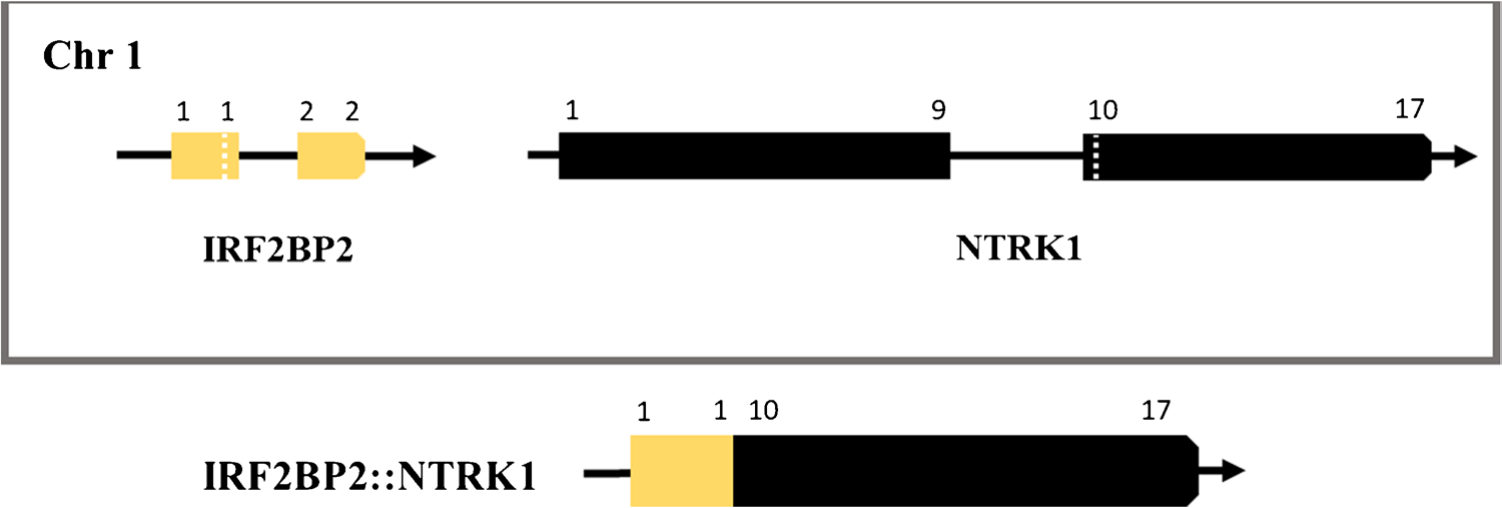
Schematic representation of the *IRF2BP2::NTRK1* gene fusion. The fusion of *IRF2BP2* (NM_182972.2, exon 1) and *NTRK1* (NM_002529.3, exon 10) was identified by RNA–based NGS panel (FusionPlex Lung v2). Exons of *IRF2BP2* are shown in yellow and *NTRK1* in black

Expanded RNA–based NGS panel FusionPlex Lung v2 was used as the gold standard for detection of gene fusions in the 62 dMLH1/*BRAF*V600Ewt CRC samples. Seven in-frame *NTRK1* fusions (Table 2) were identified (*TPM3::NTRK1*, *PLEKHA6::NTRK1* and *LMNA::NTRK1*, each in two cases, and *IRF2BP2::NTRK1* in one case), of which *IRF2BP2::NTRK1* is a novel fusion in CRC (Fig. 1). FusionPlex CTL and/or TruSight RNA Pan-Cancer NGS panels confirmed the seven *NTRK1* fusions (Supplementary Table S3). An *NTRK1* fusion was thus identified in 7/62 (11.3%) of the dMLH1/*BRAF*V600Ewt, in 7/43 (16.3%) of the dMLH1/*BRAF*V600Ewt/*MLH1*ph and in 7/30 (23.3%) of the dMLH1/*BRAF*V600Ewt/*MLH1*ph/*RAS*wt CRC samples. In addition to *NTRK1* fusions, Lung v2 NGS detected two in-frame *ALK* fusions (*EML4::ALK* in both), four in-frame *RET* fusions (*CCDC6::RET* in three cases and *NCOA4::RET* in one case) and seven in-frame *BRAF* fusions (*AGAP3::BRAF* and *TRIM24::BRAF* both in two cases, and *STARD3NL::BRAF*, *MKRN1::BRAF* and *LMTK2::BRAF* each in one case) (Table 2). Analysis by the Lung v2 NGS failed in four cases (#7, 9, 11 and 26). These four samples were reanalysed using FusionPlex CTL NGS kit, and three were found to be fusion negative, whereas one failed with this panel as well (case 7). A kinase fusion was thus identified in 20/62 (32.3%) of the dMLH1/*BRAF*V600Ewt, in 20/43 (46.5%) of the dMLH1/*BRAF*V600Ewt/*MLH1*ph and in 20/30 (66.7%) of the dMLH1/*BRAF*V600Ewt/*MLH1*ph/*RAS*wt CRC samples.

**Table 2:** Characteristics of the colorectal cancer cases with gene fusions

**Case**	**Age**	**Sex**	**Tumour site**	**Grade**	**pTNM**	**FusionPlex Lung v2 NGS**	**Idylla GeneFusion**	**Immunohistochemistry** (% of positive tumour cells)
19	83	Female	Right	Low	pT3N1bM0	*TPM3*(e7)::*NTRK1*(e10)	*NTRK1* imb	Pan-TRK: Cp + + + and M + + (100%) ALK: negative
52^a^	62	Male	Right	Low	pT3N0M1	*TPM3*(e7)::*NTRK1*(e10)	*NTRK1* imb	Pan-TRK: Cp + + and M + + (100%) ALK: negative
29	88	Female	Right	High	pT3N0M0	*LMNA*(e5)::*NTRK1*(e11)	*NTRK1* imb *ALK* imb^b^	Pan-TRK: Cp + + and Pn (100%) ALK: negative
35	65	Female	Right	Low	pT4bN0M1	*LMNA*(e4)::*NTRK1*(e10)	*NTRK1* imb *ALK* imb^c^	Pan-TRK: Cp + + + and Pn (100%) ALK: negative
25	51	Female	Right	Low	PT3N0M0	*PLEKHA6*(e21)::*NTRK1*(e10)	*NTRK1* imb	Pan-TRK: Cp + and M + + (80%) ALK: negative
50	88	Male	Right	Low	pT2N0M0	*PLEKHA6*(e21)::*NTRK1*(e10)	*NTRK1* imb	Pan-TRK: Cp + and M + + (90%) ALK: negative
59	71	Female	Left	Low	pT3N1aM0	*IRF2BP2*(e1)::*NTRK1*(e10)	*NTRK1* imb	Pan-TRK: Cp + + (100%) ALK: negative
12	72	Female	Right	Low	pT4bN0M1	*EML4*(e21*)*::*ALK*(e20)	*ALK* imb	Pan-TRK: Cp + (50%) ALK: Cp + + + (100%)
16	71	Female	Right	Low	pT2N1bM0	*EML4*(e6)::*ALK*(e20)	*ALK* specific	Pan-TRK: negative ALK: Cp + + + (100%)
24	87	Male	Right	Low	pT3N1aM0	*CCDC6*(e1)::*RET*(e12)	*RET* specific/imb *ALK* imb^d^	Pan-TRK and ALK negative
46	66	Female	Right	High	pT3N2bM0	*CCDC6*(e8)::*RET*(e12)	*ALK* imb	Pan-TRK and ALK negative
49	70	Female	Right	High	pT2N0M0	*CCDC6*(e1)::*RET*(e12)	*RET* specific/imb	Pan-TRK and ALK negative
43	83	Male	Right	High	pT3N1cM1	*NCOA4*(e9)::*RET*(e12)	Not detected	Pan-TRK and ALK negative
4	77	Female	Right	Low	pT2N2aM0	*AGAP3*(e8)::*BRAF*(e9)	Not included in the assay	Pan-TRK and ALK negative
13	62	Male	Right	Low	pT3N0M0	*AGAP3*(e13)::*BRAF*(e10)	Not included in the assay	Pan-TRK and ALK negative
38	73	Male	Right	Low	pT1N0M0	*TRIM24*(e10)::*BRAF*(e9)	Not included in the assay	Pan-TRK and ALK negative
54	90	Male	Right	High	pT3N1aM0	*TRIM24*(e9)::*BRAF*(e9)	Not included in the assay	Pan-TRK and ALK negative
31	71	Female	Right	Low	pT3N0M0	*STARD3NL*(e7)::*BRAF*(e10)	Not included in the assay	Pan-TRK and ALK negative
42	72	Female	Right	High	pT4aN0M0	*MKRN1*(e4)::*BRAF*(e11)	Not included in the assay	Pan-TRK and ALK negative
51	77	Female	Right	Low	pT3N0M0	*LMTK2*(e2)::*BRAF*(e9)	Not included in the assay	Pan-TRK and ALK negative

*Cp* cytoplasmic, *e* exon, *imb* expression imbalance, *M* membranous, *NGS* next-generation sequencing, *Pn* perinuclear. ^a^Case 52 had synchronous pT4N0M1 *KRAS* mutated sigma tumour with proficient MMR and negative pan-TRK immunostaining that had metastasised to the peritoneum. ^b^Both *NTRK1* imb and *ALK* imb were detected simultaneously using the Idylla assay. ^c^The first Idylla analysis was invalid (RNA amplification improper/inappropriate). Re-analysis was done using two separate tissue blocks, and one resulted *NTRK1* imb and the other one both *NTRK1* imb and *ALK* imb. ^d^The first Idylla analysis was invalid (RNA amplification improper/inappropriate). Re-analysis was done using the same tissue block, and the repetition resulted *RET* specific/imb and *ALK* imb simultaneously

Fusion-positive cases (*n* = 20) had significantly higher preponderance of *MLH1*ph (*P* = 0.0002) and *RAS*wt genotype (*P* < 0.0001) compared to fusion negative cases (*n* = 42), but no statistically significant differences in clinicopathological features including age, sex, pTNM stage, grade or tumour site were observed between the negative and positive cases (Supplementary Table S4). One *NTRK1*, one *ALK* and one *RET* fusion case presented distant metastases (cases 12, 35 and 43) and were all *RAS*wt. Furthermore, one *NTRK1*-rearranged case with distant metastases (case 52) had synchronous sigma tumour with proficient (p)MMR and *KRAS*G12V mutation, as was also shown to be the case for the peritoneal metastasis, whilst the *NTRK1*-rearranged tumour in colon ascendens was dMMR and *RAS*wt.

### Idylla gene fusion assay

All 62 CRC samples with dMLH1 and *BRAF*V600Ewt status were next analysed using Idylla gene fusion assay. *NTRK1* expression imbalance was identified in seven cases, which coincided with the Lung v2 NGS fusion cases (Table 2 and Supplementary Table S2). The concordance between Idylla gene fusion test *NTRK1* expression imbalances and *NTRK1* fusions detected by Lung v2 NGS was 100% (59/59 valid Idylla tests, *P* < 0.0001; 3/62 Idylla analyses were invalid). Using the NGS as a reference, the sensitivity, specificity, PPV and NPV of Idylla gene fusion test to identify expression imbalance in *NTRK1* fusion cases were 100% for each. Idylla detected the Lung v2 NGS identified *EML4*(e6)::*ALK*(e20) fusion as a specific *ALK* fusion. The second *ALK* fusion identified by the Lung v2 NGS (*EML4*(e21)::*ALK*(e20)) is not included in the set of specific fusions detected by Idylla, and it was thus detected as an *ALK* expression imbalance. However, Idylla reported seven additional *ALK* expression imbalances in cases, which were not *ALK* fusion–positive by Lung v2 NGS. Thus, concordance between Idylla *ALK* specific fusion or expression imbalance and the Lung v2 NGS was 88.1% (52/59 valid Idylla tests, *P* = 0.021), and the sensitivity, specificity, PPV and NPV were 100%, 87.7%, 22.2% and 100%, respectively. In addition, two combined *RET*–specific fusion and *RET* expression imbalance results were detected in line with the Lung v2 NGS results, whilst two *RET* fusions were not detected by the Idylla analysis (specific fusion detection not included and no *RET* expression imbalance reported). Further, one false positive *RET* expression imbalance was reported. Thus, concordance between Idylla *RET*–specific fusion and/or expression imbalance and the Lung v2 NGS was 94.9% (56/59 valid Idylla tests, *P* = 0.010), and the sensitivity, specificity, PPV and NPV were 50%, 98.2%, 66.7% and 96.4%, respectively. Initially, seven Idylla results were invalid due to improper/inappropriate RNA amplification, of which four cases were successfully re-analysed (Table 2 and Supplementary Table S2). It should be pointed out that none of the initially invalid samples in the Idylla analyses was the same as the four samples that failed with the Lung v2 NGS. In addition, some individual targets remained invalid in the Idylla analyses despite re-runs, which was especially common in the expression imbalance of *NTRK3* (12/59, 20.3%; Supplementary Table S2).

### Pan-TRK and ALK IHC

**Fig. 2:**
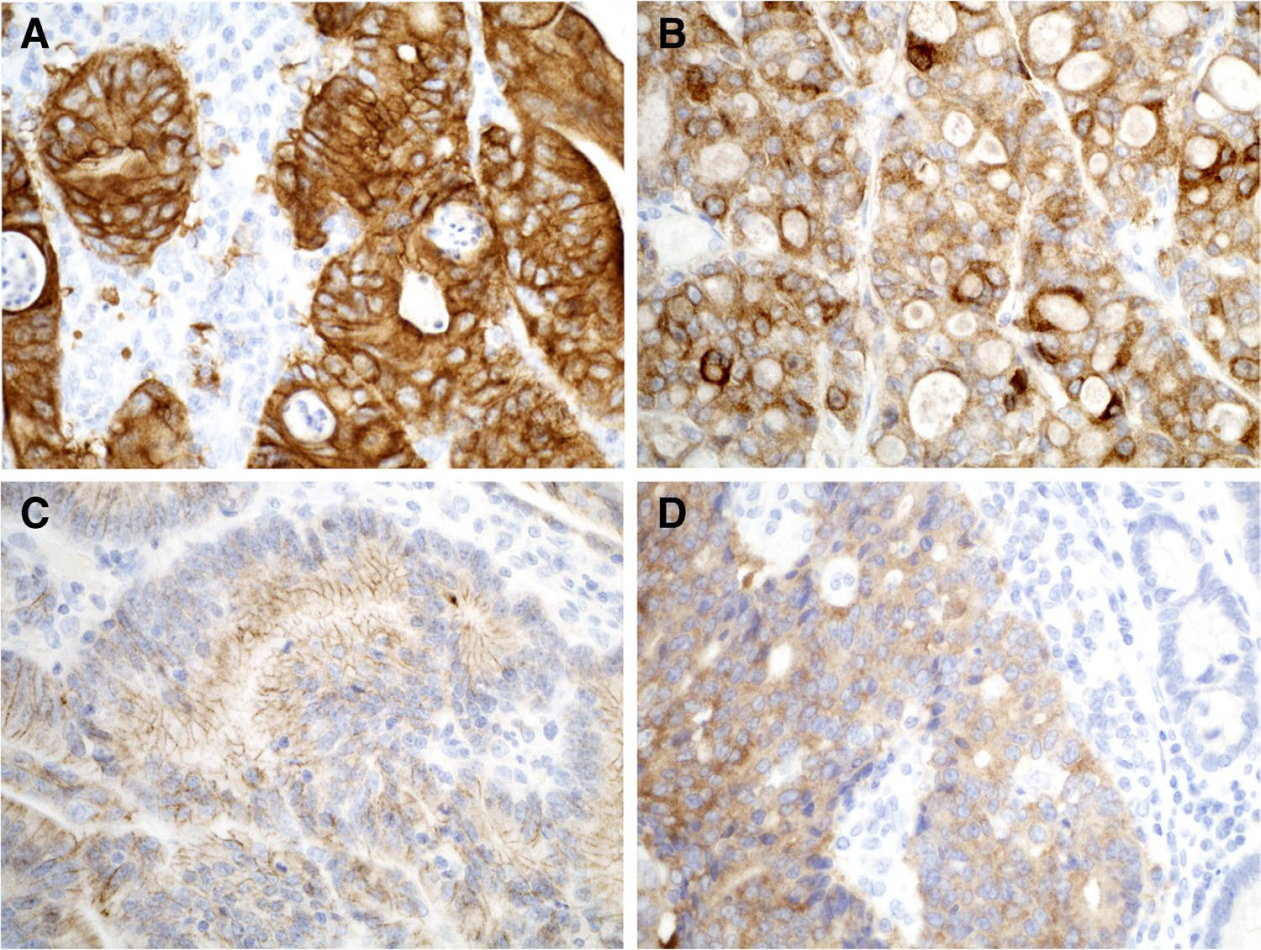
Pan-TRK (clone EPR17341) immunostaining patterns in CRC samples. **A** Strong cytoplasmic staining with moderate membranous staining (Case 19; *TPM3*::*NTRK1*). **B** Moderate cytoplasmic staining with perinuclear staining (Case 29; *LMNA*::*NTRK1*). **C** Weak cytoplasmic staining with moderate membranous staining (Case 50; *PLEKHA6*::*NTRK1).* **D** Moderate cytoplasmic staining (Case 59; *IRF2BP2*::*NTRK1*). Original magnification 400 ×

Pan-TRK IHC was used to screen for *NTRK* gene fusions in the 62 CRC samples using two antibody clones and scored in blinded manner by two observers independently. With clone EPR17341 eight and with clone A7H6R seven, CRC samples were scored as immunopositive by both observers. However, staining intensity was higher and subcellular localization clearer using the clone EPR17341 protocol and scoring results using this clone are shown in Table 2. All samples showed cytoplasmic immunopositivity with variable intensity that was relatively diffuse (50–100% of the tumour cells being positive), and four samples also expressed membranous and two perinuclear staining (Table 2, Fig. 2 and Supplementary Fig. S1). No nuclear staining was observed. The overall agreement between pan-TRK IHC (EPR17341) and NGS was 98.4% (61/62, *P* < 0.0001). Using the NGS as a reference, the sensitivity of pan-TRK (EPR17341) IHC was 100% and the specificity 98.2% with PPV of 87.5% and NPV of 100%. For the clone A7H6R, these parameters were 100%. Importantly, one EPR17341 pan-TRK IHC positive sample, expressing the lowest cytoplasmic staining intensity and being negative with clone A7H6R, did not contain an *NTRK* fusion but an *ALK* fusion (case 12) (Table 2 and Supplementary Figs. S1 and S2). This case, and the other *ALK* fusion case, showed strong cytoplasmic ALK immunopositivity, whilst the rest of the samples were completely negative (Table 2 and Supplementary Fig. S2).

## Discussion

We screened 2079 CRC resection specimens for dMLH1 and BRAFV600Ewt using IHC and identified 64 such cases of which 62 were available for this study. The BRAFV600E IHC result was confirmed in this study by MS-MLPA and Idylla NRAS-BRAF test, which both were 100% concordant. The MSI status was confirmed with Idylla MSI test. Since *NTRK* gene fusions have been shown to be enriched in this subgroup of CRC [4, 7] and solid tumours with *NTRK* fusion can be treated with larotrectinib or entrectinib [2], we were especially interested to investigate this targetable gene rearrangement using several techniques. First, RNA–based Lung v2 NGS panel was used as the gold standard, and seven (7/62, 11.3%) *NTRK1* fusions were found in our CRC cohort with dMLH1/*BRAF*V600Ewt. Westphalen et al. recently showed the prevalence of *NTRK* fusions in CRC to be 0.22% in a large real-world cohort of adult CRC cases (*n* = 34 590) using NGS–based database approach [7]. This figure is comparable to previous screening studies for *NTRK* fusions in CRC that have found the prevalence to be 0.14–0.35% using upfront NGS or IHC with NGS confirmation [4–6, 13–16]. Thus, our *NTRK* fusion frequency of 0.34% (7/2079) is at the higher end.

*NTRK1* fusions with *TPM3*, *LMNA* and *TPR* partners have been shown to be the most common ones in CRC [6, 7] and *PLEKHA6::NTRK1* fusions have also previously been described in this disease [17, 18]. Of these, we found *NTRK1* to partner with *TPM3*, *LMNA* and *PLEKHA6*. *IRF2BP2::NTRK1* fusion was found in a single case of our series, and to our knowledge, this is the first time it has been reported in CRC. Interestingly, the *IRF2BP2::NTRK1* containing tumour was the only one located to the left colon (sigma) amongst our *NTRK1* fusion–positive cases. We were, however, unable to find any *NTRK3* fusions, although Lung v2 NGS can detect a wide range of *NTRK3* fusions. This may relate to the fact that the estimated proportion of *NTRK3* fusions of all *NTRK* fusions is only 11% in CRC [7]. Strengths of our study included the use of real-life diagnostic tissue material originating from a cohort of over two thousand consecutive surgically treated primary CRC patients and the use of multiple techniques, including detection of the *NTRK1* fusions using three independent RNA–based NGS platforms. Weakness of the study was that we investigated gene fusions only in a subgroup of CRC. To this end, we may have missed a few *NTRK* fusion–positive CRCs, since a small proportion (11–19%) of *NTRK* fusions has been found in microsatellite-stable CRCs [4, 6]. Four samples failed with Lung v2 NGS, three of which were found to be fusion-negative by the FusionPlex CTL NGS analysis, whereas the fourth one failed with CTL panel as well.

Westphalen et al. found CRC to be the only cancer type in which *NTRK* fusions are associated with sporadic MSI [7]. Interestingly, Kim et al. have recently described that *NTRK* fusions in CRC develop along the serrated pathway, in which sporadic d*MLH1* is a major molecular event, and these fusions can already be present in premalignant sessile serrated lesions [19]. Additionally, several other NGS–based studies have suggested 2.6–7.3% occurrence of *NTRK* fusions in dMMR/MSI CRCs [15, 16, 18, 20, 21]. In the dMLH1*/BRAF*V600Ewt subgroup, frequency of *NTRK* fusions has been reported to be 5–28% [4, 16, 17] and in the subgroup of dMLH1*/MLH1*ph 14–19% [18, 19], which are in line with our frequencies of 11% (7/62) and 16% (7/43), respectively. Yet other studies have reported *NTRK* fusions to occur in dMLH1*/BRAF*V600Ewt/*MLH1*ph/*RAS*wt subgroup of CRC with as high frequency as 17–44% [15–18, 22] being comparable to our prevalence of 23.3% (7/30).

We also evaluated the performance of the novel fully automated Idylla gene fusion assay and pan-TRK IHC. Idylla gene fusion test was 100% specific and sensitive to detect *NTRK1* expression imbalance in the seven *NTRK1* fusion CRC cases. However, Idylla reported initially invalid result in seven cases (7/62, 11%), of which three remained invalid after re-analysis, and additional 12 isolated invalid results for *NTRK3* expression imbalance. Interestingly, the high frequency of *NTRK3* invalids might originate from promoter methylation causing loss of *NTRK3* expression, which has been reported in over 11% of CRC cases [23]. Furthermore, our study shows that specificity and sensitivity of pan-TRK IHC are optimal in CRC samples. Although specificity of this method varies between different tumour types, it has previously been reported to be 100% for pan-TRK in CRC [4, 13, 17, 24]. Pan-TRK IHC positive CRCs are characterised by cytoplasmic staining with additional positivity in other subcellular compartments in a fusion partner-dependent manner. Membranous staining has been linked to *TPM3*, *TPR* and *PLEKHA6*; perinuclear staining to *LMNA* and *MUC2* and nuclear staining to *ETV6* [6, 24–26]. We also detected moderate membranous staining along with variable intensity of cytoplasmic staining with *TPM3* and *PLEKHA6* fusion partners and perinuclear staining with *LMNA*. *IRF2BP2::NTRK1* fusion represents only 2% of all *NTRK* fusions [7], but it has been reported in lung, thyroid and prostate cancers [3]. In lung cancer, it shows cytoplasmic immunostaining [25], which was the case with our CRC sample as well.

In addition to *NTRK1* fusions, Lung v2 NGS detected two *ALK* fusions (2/62, 3.2%) in our CRC cohort, which both partnered with the most common *ALK* fusion partner *EML4* in CRC [27]. ALK IHC showed strong cytoplasmic staining in both cases, whilst the rest of the samples were completely negative. Our results thus suggest a prevalence of 0.10% (2/2079) for *ALK* fusions in CRC, which is comparable to previously published *ALK* fusion prevalence of 0.05–0.6% in CRC [27–30]. The Idylla fusion assay detected the *EML4*(e6)::*ALK*(e20) fusion as a specific fusion and the *EML4*(e21)::*ALK*(e20) fusion as an *ALK* expression imbalance. The *EML4*(e21)::*ALK*(e20) fusion with non-canonical breakpoint of *EML4* gene at exon 21 has been reported to constitute only about 2% of the *ALK*-rearrangements in non-small cell lung cancer, where *EML4*(e6)::*ALK*(e20) is the most common *EML4*::*ALK* variant [31]. The less frequent *EML4*(e21)::*ALK*(e20) variant is not covered by the Idylla’s fusion-specific detection, which is designed to catch the most relevant gene fusions in lung cancer. However, *EML4*(e21)::*ALK*(e20) fusion variant seems to be more frequent molecular event in CRC [22, 27, 29, 30]. In addition to the NGS–identified *ALK* fusions, Idylla detected seven false positive *ALK* expression imbalances. Based on our study, detection of specific *ALK* fusion seems to be a valid result, whereas all expression imbalance results need to be validated by a more specific method.

Besides *NTRK1* and *ALK* fusions, Lung v2 NGS detected four *RET* fusions (4/62, 6.5%) and seven *BRAF* fusions (7/62, 11.3%) in our CRC cohort. We found *RET* to partner with *CCDC6* in three cases and *NCOA4* in one case, both of which seem to be quite common *RET* fusion partners in CRC [32, 33]. *BRAF* fusions with partners *AGAP3*, *TRIM24* and *MKRN1* found in our study have also been previously reported to occur in CRC [15, 16, 34]. *STARD3NL::BRAF* fusion has earlier been described at least in one paediatric sarcoma [35], whereas to our best knowledge, *LMTK2::BRAF* has not been reported before in any tumour type. Two of the four Lung v2 NGS–detected *RET* fusions were detected by the Idylla assay as both *RET*–specific fusion and expression imbalance. The Idylla gene fusion test does not include *BRAF* fusions. Taken together, the Idylla platform identified two specific *RET* fusions that were in line with the NGS results, but did not report expression imbalance of two NGS–detected *RET* fusions (detection of these specific fusions is not included in the Idylla assay).

Upfront RNA–based NGS analysis is the most comprehensive, sensitive and specific method to identify gene fusions, but it is also time-consuming and requires more labour, expertise and financial resources when compared to other methods. The Idylla platform offers the fastest turnaround time with moderate cost, whilst IHC is the most affordable option. It is however clear that both Idylla expression imbalance and pan-TRK IHC results need to be validated using an alternative method, preferably an RNA–based NGS [2, 8]. To our knowledge, this is the first publication where the RUO FusionPlex Lung v2 NGS and the Idylla GeneFusion assay have been used in detecting gene fusions. As there is a tumour agnostic oncological treatment for cancer patients with an *NTRK* fusion, we would like to propose that one should screen for this gene rearrangement in CRC patients with dMLH1/*BRAF*V600Ewt/*MLH1*ph using Idylla gene fusion test or pan-TRK IHC, followed by an RNA–based NGS confirmation of the positive cases, or alternatively using upfront RNA–based NGS depending on local resources.

## Supplementary Information

Below is the link to the electronic supplementary material.

Supplementary file1 (PDF 1763 KB)

## Data Availability

The data obtained during the current study are available from the corresponding author AR on reasonable request.

## Abbreviations

ALK: Anaplastic lymphoma kinase
BRAF: V-raf murine sarcoma viral oncogene homolog B1
CRC: Colorectal cancer
CTL: Comprehensive Thyroid and Lung panel
dMMR: Deficient MMR
EMA: European Medicines Agency
FFPE: Formalin-fixed paraffin-embedded
HUH: Helsinki University Hospital
IHC: Immunohistochemistry
KRAS: Kirsten rat sarcoma viral oncogene homolog
LS: Lynch syndrome
MLH1: MutL homolog 1
MMR: Mismatch repair
MSI: Microsatellite instability
MS-MLPA: Methylation-specific multiplex ligation-dependent probe amplification
NGS: Next-generation sequencing
NPV: Negative predictive value
NRAS: Neuroblastoma ras viral oncogene homolog
NTRK: Neurotrophic tyrosine receptor kinase
pMMR: Proficient MMR
PPV: Positive predictive value
RAS: Rat sarcoma virus
RET: rearranged during transfection
RT-PCR: Reverse transcription-polymerase chain reaction
RUO: Research use only
TRK: Tropomyosin receptor kinase
wt: Wild-type
