# Supplementary material for: Gene fusions and oncogenic mutations in MLH1 deficient and BRAFV600E wild-type colorectal cancers
Source: Virchows Arch. 2022 Mar 3;480(4):807–17. doi: 10.1007/s00428-022-03302-x (PMC9023403; doi:10.1007/s00428-022-03302-x)
Supplement: Supplementary file 1 — Supplementary file1 (PDF 1763 KB) [file 428_2022_3302_MOESM1_ESM.pdf]

Virchows Archiv

Supplementary Information

for

Gene fusions and oncogenic mutations in MLH1 deficient and *BRAF*V600E wild-type colorectal cancers

Iiris Ukkola[1,2], Pirjo Nummela[2], Mia Kero[1], Hanna Tammio[3], Jenni Tuominen[4], Veli Kairisto[4], Markku Kallajoki[5], Caj Haglund[6,7], Päivi Peltomäki[8], Soili Kytölä[3] and Ari Ristimäki[1,2]*

[1]Department of Pathology, HUSLAB, HUS Diagnostic Center, Helsinki University Hospital and University of Helsinki, Helsinki, Finland

[2]Applied Tumor Genomics Research Program, Research Programs Unit, Faculty of Medicine, University of Helsinki, Helsinki, Finland

[3]Department of Genetics, HUSLAB, HUS Diagnostic Center, Helsinki University Hospital and University of Helsinki, Helsinki, Finland

[4]Department of Genomics, Laboratory of Molecular Haematology and Pathology, Turku University Central Hospital, Turku, Finland

[5]Department of Pathology, University of Turku and Turku University Hospital, Turku, Finland

[6]Department of Surgery, University of Helsinki and Helsinki University Hospital, Helsinki, Finland

[7]Translational Cancer Medicine Research Program, Research Programs Unit, Faculty of Medicine, University of Helsinki, Helsinki, Finland

[8]Department of Medical and Clinical Genetics, University of Helsinki, Helsinki, Finland

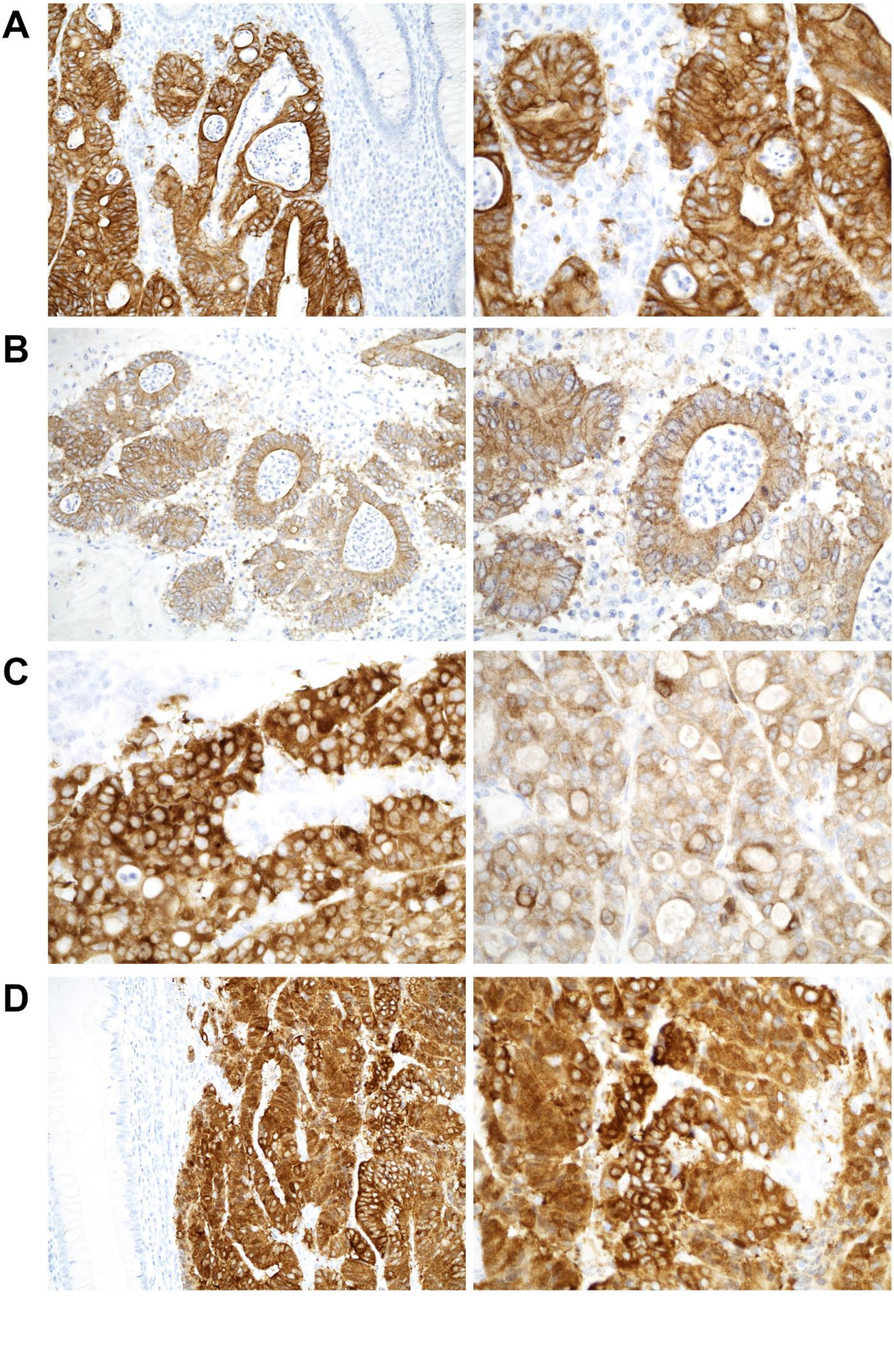
A
B
C
D

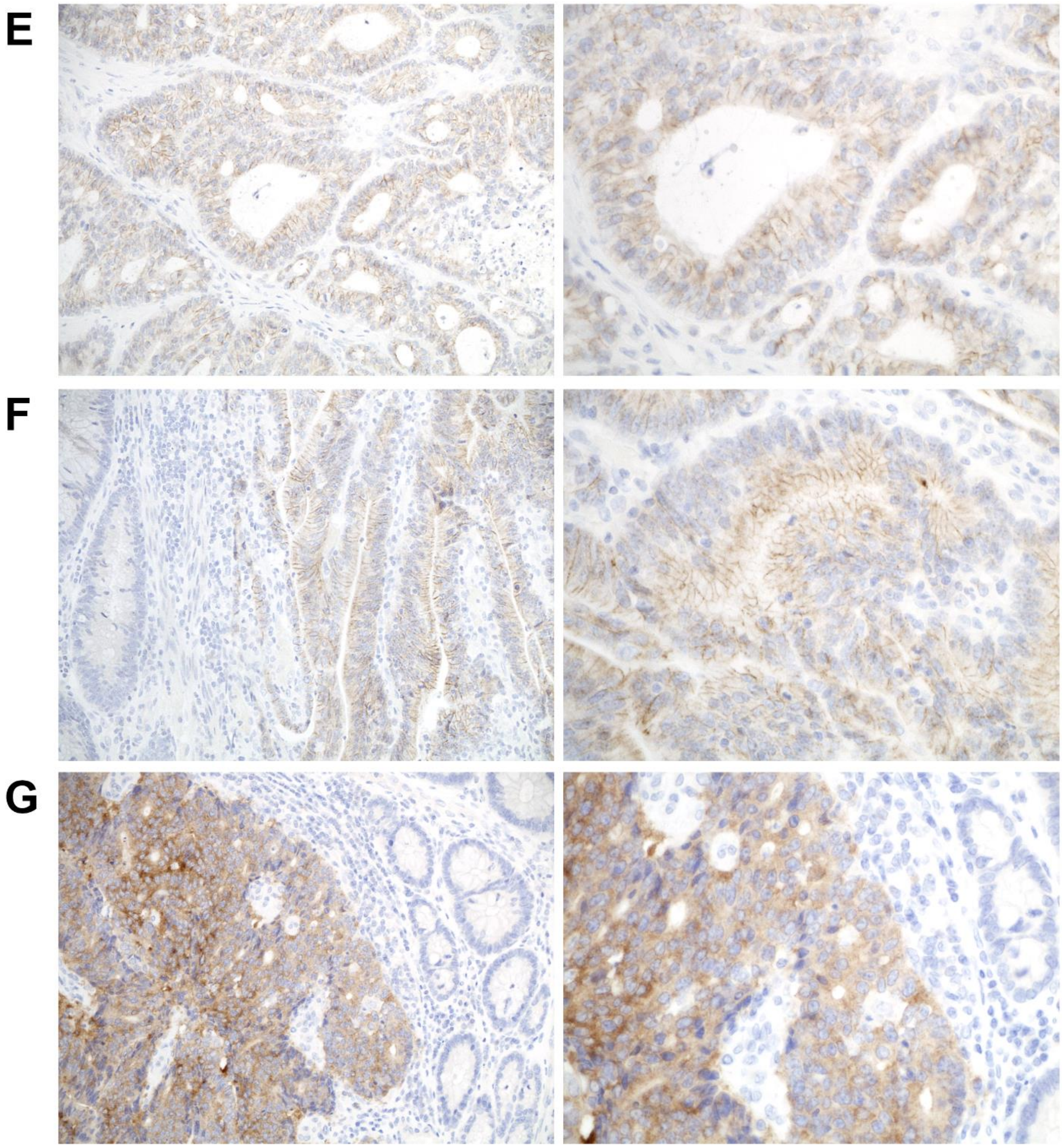

Supplementary Figure S1. Pan-TRK (clone EPR17341) immunostaining patterns in CRC samples. (**A**) Strong cytoplasmic staining with moderate membranous staining (Case 19; *TPM3::NTRK1*). (**B**) Moderate cytoplasmic staining with moderate membranous staining (Case 52; *TPM3::NTRK1*). (**C**) Moderate cytoplasmic staining with perinuclear staining (Case 29; *LMNA::NTRK1*). (**D**) Strong cytoplasmic staining with perinuclear staining (Case 35; *LMNA::NTRK1*). (**E**) Weak cytoplasmic staining with moderate membranous staining (Case 25; *PLEKHA6::NTRK1*). (**F**) Weak cytoplasmic staining with moderate membranous staining (Case 50; *PLEKHA6::NTRK1*). (**G**) Moderate cytoplasmic staining (Case 59; *IRF2BP2::NTRK1*). Original magnification x200 on the left side and x400 on the right.

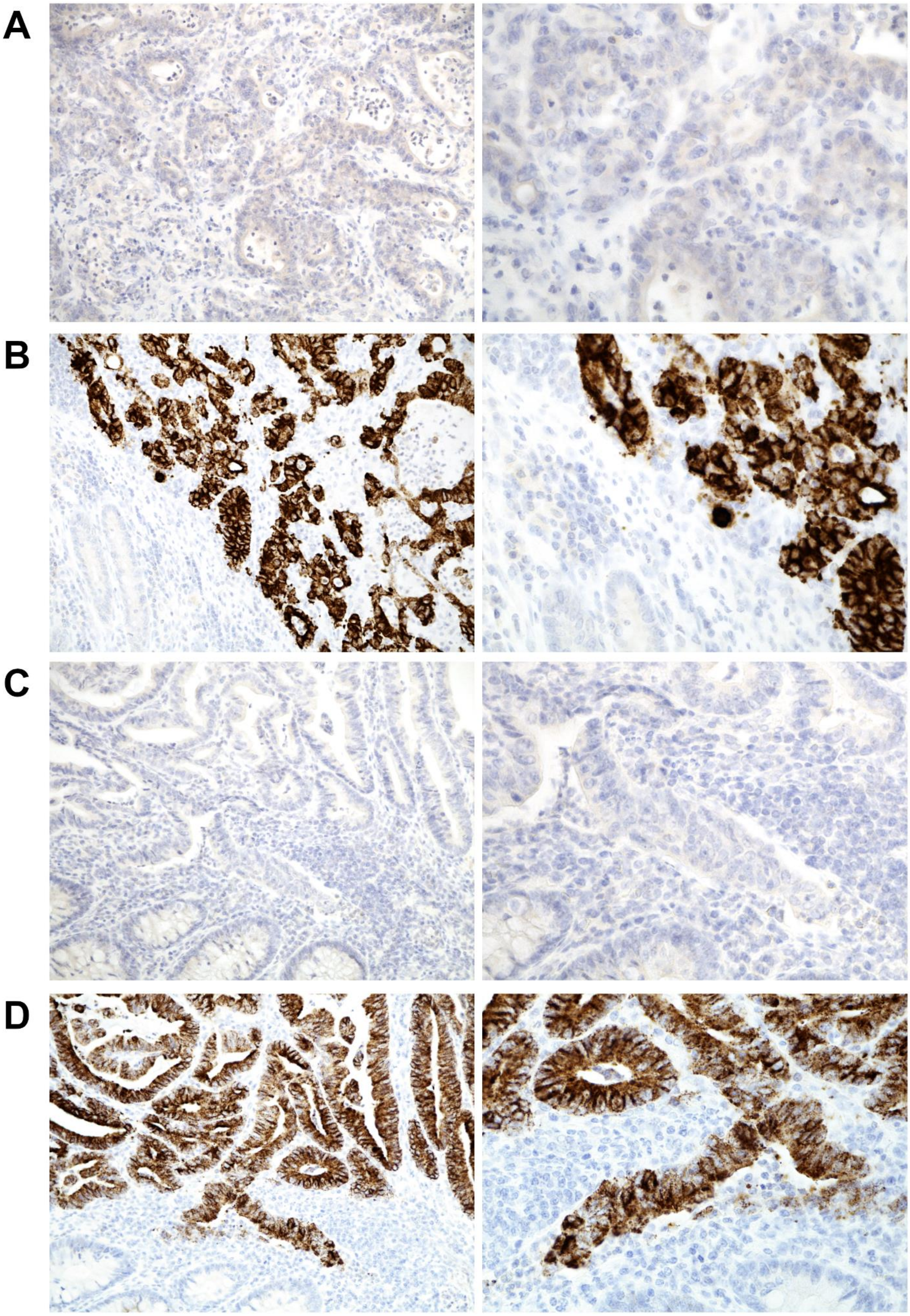
A
B
C
D

Supplementary Figure S2. ALK (clone 5A4) and pan-TRK (clone EPR17341) immunostaining patterns in CRC samples. (**A**) Weak cytoplasmic staining in pan-TRK IHC (Case 12; *EML4::ALK*). (**B**) Strong cytoplasmic staining in ALK IHC (Case 12; *EML4::ALK*). (**C**) Negative Pan-TRK IHC (Case 16; *EML4::ALK*). (**D**) Strong cytoplasmic staining in ALK IHC (Case 16; *EML4::ALK*). Original magnification x200 on the left side and x400 on the right.

Supplementary Table S1. Reference sequences used in the NGS fusion analyses

	**FusionPlex Lung v2 NGS (GRCh37)**
AGAP3	NM_031946.6
ALK	NM_004304.4
BRAF	NM_004333.4
CCDC6	NM_005436.4
EML4	NM_019063.4
IRF2BP2	NM_182972.2
LMNA	NM_005572.3 (10 exons, 572 AAs)
LMTK2	NM_014916.3
MKRN1	NM_013446.3
NCOA4	NM_005437.3
NTRK1	NM_002529.3
PLEKHA6	NM_014935.4
RET	NM_020630.4
STARD3NL	NM_032016.3
TPM3	NM_153649.3 (8 exons, 248 AAs)
TRIM24	NM_003852.3

	**FusionPlex CTL NGS (GRCh37)**	**TruSight NGS (GRCh38)**
IRF2BP2	NM_182972.2	NM_182972.3
LMNA	NM_005572.3 (10 exons, 572 AAs)	NM_170707.4 (12 exons, 664 AAs)
NTRK1	NM_002529.3	NM_002529.4
PLEKHA6	NM_014935.4	NM_014935.5
TPM3	NM_153649.3 (8 exons, 248 AAs)	NM_152263.4 (10 exons, 285 AAs)

CTL: Comprehensive Thyroid and Lung, GRC: Genome Reference Consortium, NGS: next-generation sequencing

Supplementary Table S2. Idylla and MS-MLPA results in 62 deficient MLH1/*BRAF*V600E wild-type colorectal cancers

**Case**	**Tumor cells %**	**Tissue area mm²**	**Idylla GeneFusion**	***ALK* specific**	***ALK* imb**	***ROS1* specific**	***ROS1* imb**	***RET* specific**	***RET* imb**	***MET* exon 14 skipping**	***NTRK1* imb**	***NTRK2* imb**	***NTRK3* imb**	**Idylla KRAS**	**Idylla NRAS-BRAF**	***MLH1*ph**
1	70	150	not detected											Q61R/L		yes
2[a]	40	280	not detected											A146P/T/V		no
3	30	160	not detected											G12D		no
4	50	150	not detected										invalid			yes
5	80	70	not detected											Q61K		yes
6	60	100	not detected													no
7	60	250	not detected										invalid	G12D		yes
8	50	100	not detected										invalid	G12D		yes
9	40	300	not detected													no
10	40	140	not detected													yes
11	50	270	not detected													NA
12	60	300	detected		detected											yes
13	50	120	not detected													yes
14	50	140	not detected											G12D		yes
15[b]	50	280	invalid	invalid		invalid		invalid		invalid	invalid	invalid	invalid			yes
16	30	50	detected	detected												yes
17	70	70	not detected										invalid	G12V		yes
18	70	300	not detected										invalid		Q61R	yes
19	40	60	detected								detected					yes
20	70	40	not detected													yes
21	80	30	not detected										invalid			no

22	70	40	not detected										invalid	A146P/T/V		no
23	80	130	not detected													yes
24[c]	70	140	detected		detected			detected	detected				invalid			yes
25	60	160	detected								detected		invalid			yes
26	60	200	not detected											G12D		no
27	40	90	not detected											A146P/T/V		no
28	70	75	not detected													yes
29	70	300	detected		detected						detected					yes
30	50	130	not detected											Q61K		yes
31	30	60	not detected													yes
32	40	150	detected		detected				detected							yes
33	30	280	detected		detected											no
34	50	300	detected		detected									Q61K		yes
35[d]	40	150	detected		detected						detected					yes
36	50	220	not detected													yes
37	50	140	not detected											A146P/T/V		yes
38	30	50	not detected													yes
39	60	100	not detected													no
40	40	50	not detected	invalid										G12D		no
41	40	120	not detected													no
42	30	150	not detected										invalid			yes
43	60	160	not detected													yes
44	50	100	not detected													no
45	40	150	not detected											G13D		no
46	50	300	detected		detected			invalid								yes
47	70	130	not detected											G12D		no
48	70	170	not detected											Q61R/L		yes
49	70	50	detected		invalid			detected	detected				invalid			yes
50	60	50	detected								detected					yes
51	60	50	not detected													yes

52	50	150	detected								detected					yes
53	60	300	not detected													yes
54	70	75	not detected													yes
55[a]	50	150	not detected										invalid		G12C	no
56[b]	70	200	invalid	invalid	invalid	invalid	invalid	invalid	invalid	invalid	invalid	invalid	invalid		Q61R	yes
57	40	290	not detected													yes
58	20	120	not detected													no
59	50	100	detected								detected					yes
60	40	150	not detected											G12D		yes
61	40	100	not detected													yes
62[b]	50	300	invalid	invalid	invalid	invalid	invalid	invalid	invalid	invalid	invalid	invalid	invalid	G12V		no

imb: expression imbalance, MS-MLPA: methylation-specific multiplex ligation-dependent probe amplification, ph: promoter hypermethylation

[a]The first Idylla analysis was invalid (RNA amplification improper/inappropriate). Re-analysis was done using the same tissue block, and the repetition resulted not detected.

[b]The first Idylla analysis was invalid (RNA amplification improper/inappropriate). Re-analysis was done using the same tissue block, and the result remained as invalid.

[c]The first Idylla analysis was invalid (RNA amplification improper/inappropriate). Re-analysis was done using the same tissue block, and the repetition resulted *RET* specific/imb and *ALK* imb simultaneously.

[d]The first Idylla analysis was invalid (RNA amplification improper/inappropriate). Re-analysis was done using two separate tissue blocks, and one resulted *NTRK1* imb and the other one both *NTRK1* imb and *ALK* imb.

Supplementary Table S3. Colorectal cancer cases with *NTRK1* fusions analysed by three different RNA-based NGS panels

Case	FusionPlex Lung v2 NGS (GRCh37)	FusionPlex CTL NGS (GRCh37)	TruSight NGS (GRCh38)
19	*TPM3*(e7)::*NTRK1*(e10)	*TPM3*(e7)::*NTRK1*(e10)	*TPM3*(e8)::*NTRK1*(e10)
52	*TPM3*(e7)::*NTRK1*(e10)	*TPM3*(e7)::*NTRK1*(e10)	Not determined
30	*LMNA*(e5)::*NTRK1*(e11)	*LMNA*(e5)::*NTRK1* (e11)	*LMNA*(e4)::*NTRK1*(e11)
36	*LMNA*(e4)::*NTRK1*(e10)	*LMNA*(e4)::*NTRK1*(e10)	*LMNA*(e4)::*NTRK1*(e10)
25	*PLEKHA6*(e21)::*NTRK1*(e10)	*PLEKHA6*(e21)::*NTRK1*(e10)	*PLEKHA6*(e21)::*NTRK1*(e10)
50	*PLEKHA6*(e21)::*NTRK1*(e10)	*PLEKHA6*(e21)::*NTRK1*(e10)	*PLEKHA6*(e21)::*NTRK1*(e10)
59	*IRF2BP2*(e1)::*NTRK1*(e10)	*IRF2BP2*(e1)::*NTRK1*(e10)	*IRF2BP2*(e1)::*NTRK1*(e10)

CTL: Comprehensive Thyroid and Lung, GRC: Genome Reference Consortium, NGS: next-generation sequencing

Supplementary Table S4. Comparison of clinicopathological characteristics of fusion positive and fusion negative deficient MLH1/*BRAF*V600E wild-type CRCs (n=62)

		Fusion (n=20)	No fusion (n=42)	P-value[a]
Age (years)	Median (range)	72 (51-90)	70 (36-91)	0.05
Sex	Female	13 (65.0%)	24 (57.1%)	0.17
	Male	7 (35.0%)	18 (42.9%)	
Tumour site	Left colon	1 (5.0%)	8 (19.0%)	0.25
	Right colon	19 (95%)	34 (81.0%)	
Grade	Low-grade	14 (70.0%)	29 (69.0%)	1.00
	High-grade	6 (30.0%)	13 (31.0%)	
pT	T1-T2	5 (25.0%)	8 (19.0%)	0.74
	T3-T4	15 (75.0%)	34 (81.0%)	
pN	N0	12 (60.0%)	28 (66.7%)	0.78
	N1-N2	8 (40.0%)	14 (33.3%)	
M	M0	16 (80.0%)	34 (81.0%)	1.00
	M1	4 (20.0%)	8 (19.0%)	
*MLH1*ph[b]	Present	20 (100%)	23 (56.1%)	0.0002
	Absent	0 (0.0%)	18 (43.9%)	
RAS mutation	Present	0 (0.0%)	23 (54.8%)	<0.0001
	Absent	20 (100%)	19 (45.2%)	

CRC: colorectal cancer, T: tumour, N: nodes, M: metastases, ph: promoter hypermethylation
[a]Mann-Whitney U-test (numerical variables) or Fisher's exact test (categorical variables).
[b]One fusion negative case (#11) was not available for the analysis and thus n=61.
